# Optimising trial designs to identify appropriate antibiotic treatment durations

**DOI:** 10.1186/s12916-019-1348-z

**Published:** 2019-06-21

**Authors:** Koen B. Pouwels, Mo Yin, Christopher C. Butler, Ben S. Cooper, Sarah Wordsworth, A. Sarah Walker, Julie V. Robotham

**Affiliations:** 1 Health Econonomics Research Centre, Nuffield Department of Population Health, University of Oxford, Oxford, UK; 2 Modelling and Economics Unit, National Infection Service, Public Health England, London, UK; 3 Department of Health Sciences, Global Health, University Medical Centre Groningen, University of Groningen, 9713 GZ Groningen, The Netherlands; 4 Mahidol Oxford Tropical Medicine Research Unit, Faculty of Tropical Medicine, Mahidol University, Bangkok, Thailand; 5 Division of infectious disease, University Medicine Cluster, National University Hospital, Singapore, Singapore; 6 The National Institute for Health Research Health Protection Research Unit in Healthcare Associated Infections and Antimicrobial Resistance at the University of Oxford, Oxford, UK; 7 Nuffield Department of Primary Care Health Sciences, University of Oxford, Oxford, UK; 8 Nuffield Department of Medicine, Centre for Tropical Medicine and Global Health, University of Oxford, Oxford, UK; 9 National Institute for Health Research Oxford Biomedical Research Centre, John Radcliffe Hospital, University of Oxford, |Oxford, UK; 10 MRC Clinical Trials Unit at University College London, London, UK

**Keywords:** Antimicrobial resistance, Design, Randomised trial, Duration of therapy, Antibiotics, Bayesian, Frequentist

## Abstract

**Background:**

For many infectious conditions, the optimal antibiotic course length remains unclear. The estimation of course length must consider the important trade-off between maximising short- and long-term efficacy and minimising antibiotic resistance and toxicity.

**Main body:**

Evidence on optimal treatment durations should come from randomised controlled trials. However, most antibiotic randomised controlled trials compare two arbitrarily chosen durations. We argue that alternative trial designs, which allow allocation of patients to multiple different treatment durations, are needed to better identify optimal antibiotic durations. There are important considerations when deciding which design is most useful in identifying optimal treatment durations, including the ability to model the duration–response relationship (or duration–response ‘curve’), the risk of allocation concealment bias, statistical efficiency, the possibility to rapidly drop arms that are clearly inferior, and the possibility of modelling the trade-off between multiple competing outcomes.

**Conclusion:**

Multi-arm designs modelling duration–response curves with the possibility to drop inferior arms during the trial could provide more information about the optimal duration of antibiotic therapies than traditional head-to-head comparisons of limited numbers of durations, while minimising the probability of assigning trial participants to an ineffective treatment regimen.

## Background

Bacteria are increasingly able to resist antibiotic treatment, resulting in increased morbidity, deaths and costs worldwide [1–5]. Antibiotic use is an important driver of the development and spread of antimicrobial resistance [6]. Selective pressure can be reduced by minimising antibiotic prescribing for conditions for which antibiotics are often unnecessary [7–10]. Moreover, the duration of antibiotic courses can often be reduced without significantly compromising cure rates [11–15]. Shortening antibiotic duration can have a large impact on reducing exposure of bacteria to antibiotics, including bacteria carried asymptomatically [14]. Antibiotics are by far the most prescribed drugs for children, with more than 60 million systemic antibiotics dispensed annually in the US outpatient setting alone [16], and are amongst the most frequently prescribed drugs for adults [17].

An important challenge is that, for many infectious conditions, the optimal antibiotic course length remains unclear. Courses should be long enough to treat infections effectively, yet short enough to reduce the incidence of side effects and the development and spread of antibiotic resistance. The continued need for antibiotics can be assessed with daily reviews for inpatients [18]; however, in practice, such reviews are not always performed or acted upon and antibiotics are often continued in order to complete currently recommended course durations [19]. Further, in the outpatient or primary care setting, continued assessment of patients initiated on antibiotics is not feasible [14]. Therefore, it is especially important to have strong evidence about optimal treatment durations in these settings.

Because observational studies comparing different antibiotic durations are potentially confounded by unmeasured patient factors influencing the need for prolonged treatment, evidence about the optimal treatment duration should, where possible, come from randomised controlled trials (RCTs). However, antibiotic durations for several infections managed in primary care, such as prostatitis, are not guided by RCT evidence on optimal treatment duration [20, 21]. Where treatment durations have been compared in RCTs, in most cases, two treatment durations were selected for comparison, both of which lacked a clear scientific rationale [11, 12]. Whilst RCTs designed in this way can be useful, there are disadvantages to this approach.

Herein, we discuss the main issue with conventional two-arm trial designs, how to assess the ‘optimal’ antibiotic treatment duration, four alternative trial designs that can estimate much needed duration–response relationships (subsequently denoted duration–response curves), and which of these designs has the most desirable properties.

### Issue with conventional designs

Historically, RCTs have an experimental and comparator arm, or two contrasting experimental arms [22]. An issue with conventional two-arm trials is that they are unlikely to identify optimal treatment durations, potentially leading to suboptimal clinical practice. An approach that is more likely to identify optimal treatment durations is the modelling of duration–response curves.

**Fig. 1:**
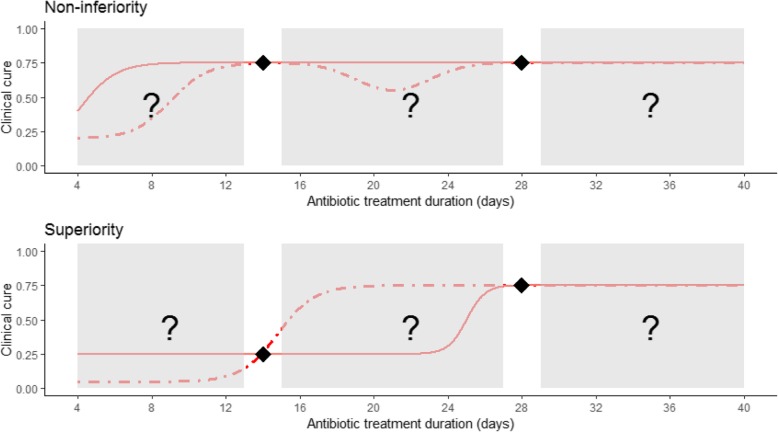
Duration–response curves corresponding to an intention-to-treat analysis. Diamonds show hypothesised event rates for the two randomised groups as designed. The solid and dot-dashed lines show different hypothesised duration–response curves that are compatible with those hypothesised event rates. This figure illustrates that conventional randomised controlled trials that compare two different durations do not provide information about other durations, especially if one duration is clearly superior to the other

In the specific example of prostatitis, we could design a conventional RCT comparing, for example, treatment durations of 14 versus 28 days. Depending on whether the trial is designed to show superiority or non-inferiority, the trial answers the question ‘is 14 days of antibiotic treatment for prostatitis as good as/worse/better than 28 days of treatment?’ (Fig. 1). However, this does not answer the more important question of ‘what is the optimal antibiotic treatment duration for prostatitis?’ The dot-dashed line in the top panel could occur if there is some non-compliance with the shorter duration as randomised, because patients are not cured (e.g. may still have persisting minor symptoms, which might then relapse without further antibiotic treatment) at the end of their assigned duration (and hence more patients end up receiving the standard duration (28 days) despite being randomised to a shorter duration). In practice, not all patients will require the same duration and, at the population level, the proportion of patients that are not cured will likely decrease with increasing assigned duration, as will the proportion with non-compliance, creating the dot-dashed line in the top panel.

### How to estimate the ‘optimal duration’?

Selecting the optimum treatment duration depends on the outcomes that are deemed important, which are often measures of cure (treatment effectiveness), either in the short-term or medium- to long-term such as prevention of relapse/recurrence. Secondary outcomes usually relate to side effects and, sometimes, to the development of resistance. The fact that cure rates can generally be hypothesised to increase with duration until reaching an asymptote creates a delicate balance between maximising efficacy and minimising adverse consequences.

The Desirability of Outcome Ranking/Response Adjusted for Duration of Antibiotic Risk (DOOR/RADAR) trial design has recently been proposed as a method to formally combine clinical outcomes and treatment duration into a single composite outcome [23]. However, in its ranking, this approach implicitly assumes that the shorter of two durations is beneficial when other patient or clinical outcomes are identical [24]. This unverified strong assumption could lead to demonstration of non-inferiority using DOOR/RADAR when conventional trial designs may show that shorter durations are not non-inferior [24].

In situations where the optimal decision regarding the best treatment depends on various endpoints, maximising a utility function (or minimising a loss function), a decision-theoretic Bayes (or full Bayesian) approach provides an intuitive solution [25, 26]. A recent Bayesian response-adaptive randomised trial evaluating the usefulness of gepotidacin for the treatment of patients with Gram-positive acute bacterial skin and skin structure infections used a utility function to determine the optimal treatment dose [25]. The dose–response-for-cure rate was modelled using a normal dynamic linear model with the parameter evolution described by a Gaussian random walk, while the dose–response-for-the-discontinuation rate was modelled with a two-parameter logistic model assuming a monotonic change [25]. The cure rate component and the treatment discontinuation component were combined multiplicatively to yield the final utility [25]. An advantage of using a utility function is that the trade-offs between the different components are made explicit and quantified. This approach provides the answer we really want to know, namely ‘what is the optimal treatment duration taking into account the trade-offs between efficacy and safety and antibiotic resistance development?’ However, given the difficulty in devising a generally acceptable utility function and the computational complexities, the decision-theoretic Bayes approach using a utility function is rarely used [26]. A problem with applying the decision-theoretic approach in medicine is that there are many decision-makers at different stages, including policy-makers, physicians and patients, who likely have different opinions and utility functions [27]. For example, different individuals may not agree that the cure and the discontinuation rates can be combined multiplicatively, and therefore also question the validity of the response-adaptive changes in the trial.

Given these and other difficulties with implementing a decision-theoretic approach [28], it may be more practical to model separate duration–response curves for efficacy, antibiotic resistance and toxicity during the trial, and combine the information from the different duration–response curves with additional information, such as costs or estimated longer term influence on resistance, into a decision analytic framework [29]. Optimal durations can then be assessed for various prior opinions and utility functions of the different stakeholders.

The optimal treatment duration may differ depending on host- or pathogen-specific characteristics. Formally, this can be tested by including different subgroup-specific interaction terms in models relating duration to response [29]. This could allow stratified medicine, enabling different optimal durations to be identified depending on key patient characteristics.

## Main characteristics of alternative RCT designs

Recently, various groups have suggested that fixed or adaptive trials could be used to assess which treatment duration should be recommended [29, 30]. Herein, we discuss multi-arm designs to model the duration–response curve, including (1) a Bayesian response-adaptive randomisation (RAR) design [31]; (2) a play-the-winner design [30, 32]; (3) a fixed duration design [29]; and (4) a drop-the-loser design.

All four designs could be used in combination with flexible regression modelling strategies to model the duration–response curve, such as fractional polynomials, or in the case of frequent reassessment of the duration–response curve, normal dynamic linear models [25, 29, 33]. To account for uncertainty about the structural form of the duration–response curve generating the data, model averaging can be used [34, 35].

The main differences between the four alternative designs and the typical two-arm frequentist randomised trial are listed in Table 1.

**Table 1:** Main characteristics of conventional two-arm and alternative multi-arm duration trial designs

	Two-arm duration design	Bayesian multi-arm, response-adaptive randomisation design	Multi-arm, play-the-winner design	Multi-arm, fixed duration design	Multi-arm, drop-the-loser duration design
Obtain information on treatment durations	Only two considered durations	Can model the entire duration–response curve, including estimates for durations that were not used	Can model the entire duration–response curve, including estimates for durations that were not used	Can model the entire duration–response curve, including estimates for durations that were not used	Can model the entire duration–response curve, including estimates for durations that were not used
Randomisation probabilities	Fixed	Variable and possibility to drop arms	Fixed, but with possibility to select shortest duration with high probability of being non-inferior for comparison with standard duration	Fixed	Fixed, but with possibility to drop arms
Higher chance for patients to be randomised to better-performing treatment arm	No	Yes	Yes	No	No, but poor performing arms can be dropped

The Bayesian RAR design allows allocation of a greater proportion of future patients to treatment durations that have performed well at the interim analysis based on posterior predictive probabilities [31]. Unless the posterior predictive probability is too low (arm should be dropped) or sufficiently high (the arm may be selected for the next phase of testing or selected as the optimal treatment), the updated randomisation probability is typically proportional to the predictive probability of success for the experimental relative to the control arm [28].

The play-the-winner design includes an option to continue with the shortest duration that has a posterior predictive probability (or a frequentist test statistic) above a pre-defined threshold compared to the standard duration, based on the assumption that shorter durations will be better in terms of risk of antibiotic resistance and toxicity.

The fixed duration design has been recently proposed by Quartagno et al. [29]. In contrast to the other designs, this is not an adaptive trial design, but focusses on gaining sufficient information to accurately model the ‘duration–response’ relationship.

The drop-the-loser design uses stopping boundaries to determine whether experimental treatments should be dropped early by comparing each to the standard (assumed maximum duration) treatment. At interim analysis, it can be decided to drop clearly inferior treatment arms compared to the standard duration based on Bayesian posterior predictive probabilities [26], or based on other predefined stopping criteria using a frequentist test-statistic [32, 36].

## Which design has the most desirable properties?

Important considerations when designing a randomised trial to identify optimal antibiotic treatment durations include (1) the ability to accurately estimate the duration–response curve, (2) the ability to minimise the risk of bias, (3) the possibility to drop poorly performing arms, and (4) statistical efficiency.

### Estimating duration–response curves

A major benefit of estimating a duration–response curve is that the effects of durations not included in the trial can also be estimated provided that there is sufficient data from neighbouring durations. This applies to all four alternative RCT designs considered here.

However, a potential issue with adaptive designs that preferentially assign patients to better performing arms (RAR and play-the-winner designs) is that this may hamper proper evaluation of the complete duration–response curve due to an insufficient number of patients receiving different durations. One could prevent this issue by assigning patients preferentially to informative treatment durations, i.e. durations that would increase the precision in an area of the curve, or by setting a threshold to the maximum imbalance in randomisation probabilities. Nevertheless, in practice, it may be more feasible to use designs with fixed randomisation probabilities (fixed duration design), potentially with the option to drop arms that are clearly inferior to the standard duration (drop-the-loser design).

Subgroup-specific duration–response curves could be obtained by including interaction terms for pre-specified subgroups such as immunocompromised patients who may require longer antibiotic therapy. With RAR (all designs except the fixed duration design), changes in allocation ratios can theoretically be based on the duration–response curve within subgroups in the presence of a subgroup effect, making the trial statistically less efficient. However, it is difficult to identify subgroup effects during a trial given the lower power to detect them, and these are usually only assessed at the end of a trial. Therefore, designs which drop arms or allocate proportionately fewer patients to some arms on a population level basis (i.e. using results from the trial as a whole), may end up without sufficient information to assess whether the optimal duration varies across important subgroups.

### Risk of bias

An important challenge that applies to all alternative RCT designs comparing multiple antibiotic treatment durations is the difficulty in blinding clinicians and patients. Where a perfectly matching placebo is available and instructions are provided about the order of taking preparations, blinding is theoretically possible, yet, in practice, such a placebo is difficult/expensive to make. Therefore, duration RCTs are often open-label. When using an open-label design that preferentially allocates patients to specific durations with better outcomes (RAR design), clinicians will be able to determine, during the trial, that these durations are associated with better outcomes, thereby increasing the risk of allocation concealment (selection) bias. This knowledge can change which patients get randomised in the trial and how endpoints will subsequently be assessed. The other designs all reduce the risk of selection bias because clinicians cannot alter the selection of patients based on observed changes in allocation probabilities for these designs.

It is often cautioned that calendar time trends – which are common with infectious diseases – may introduce bias when using RAR [30, 37]. However, one can take advantage of the fact that randomisation probabilities are not constantly changing with most RAR designs. A calendar time-stratified analysis, with equal randomisation probabilities within each stratum, eliminates potential time-trend bias [38]. A larger sample size is needed with such stratified analyses, but it is important to avoid trying to gain small improvements in efficiency at the cost of introducing bias [38]. While the fixed duration design is not vulnerable to time trends due to its design, the RAR and play-the-winner design require a less efficient calendar time-stratified analysis to avoid this type of bias. When considering a drop-the-loser design one should avoid comparison of patients assigned to the dropped duration with patients that were randomised to other arms after dropping the clearly inferior arm to avoid this bias. However, this may not be problematic given that there was already enough information to deem the duration clearly inferior.

An issue encountered with all antibiotic duration trials is potential non-compliance.Non-compliance can provide a distorted picture of the efficacy of treatment durations when performing an intention-to-treat (ITT) analysis (Fig. 1). In an ITT analysis, patients are analysed according to their assigned duration, regardless of whether they actually received that duration. ITT analyses provide unbiased estimates of effectiveness, i.e. the real-world impact of the intention to receive one versus another duration, assuming that the type of non-compliance that occurred in the trial would generalise outside the trial. In situations where non-compliance reduces the difference in treatment received between two arms being compared, an ITT analysis is not conservative for a test of non-inferiority. The effect of non-compliance, which is likely not completely random, can be taken into account using instrumental variable approaches and/or g-methods as described in more detail by Berry et al. [27] and Hernan et al. [39].

### Dropping poorly performing arms

For all designs, including fixed trial designs, continuous response monitoring for serious and unexpected adverse events or lack of efficacy of certain durations by an independent data monitoring committee can ensure that patients are protected from being randomised to an unsafe arm [40]. For adaptive designs, futility stopping criteria are defined at the planning stage. This can be done for both frequentist and Bayesian trials and would provide statistical rules to help the data monitoring committee decide whether an arm should be dropped [27, 28, 32]. After dropping an arm, follow-up will continue for patients assigned to this duration. The advantage of having the option to drop poorly performing arms (drop-the-loser design) is that it potentially reduces the number of patients allocated to unfavourable antibiotic durations. This is not only ethically desirable, but may also convince more patients to participate in a trial.

### Statistical efficiency and sample size

In the recent proposal for the fixed duration design [29], simulations showed that a sample size of 500 patients divided into 5–7 equidistant arms was sufficient to estimate the duration–response curve within a 5% error margin in 95% of the simulations, suggesting that a trial using similar methodology is feasible in practice [29]. Similar simulations focusing on the numbers needed to estimate duration–response curves for the other designs do not yet exist. In general, using standard pairwise comparisons, the more arms included, the greater the sample size, but it is not clear that such pairwise comparisons are ideal for determining optimal treatment duration.

The main reason for the increasing interest in adaptive trial designs (all designs, except the fixed duration design) may be that, under some circumstances, adaptive designs are statistically more efficient than fixed trial designs [32, 37, 38, 41, 42]. However, as mentioned earlier, if patients are preferentially allocated to the best performing arms, the precision around other durations of the duration–response curve will be reduced [29]. In addition, as discussed above, to prevent bias due to time trends, stratified analysis is recommended [38], which requires a larger sample size than the potentially biased un-stratified analysis, the latter often being used in simulations comparing response-adaptive and fixed duration designs [38, 41, 42].

### The verdict

Given the considerations laid out above, the fixed duration and the drop-the-loser duration designs theoretically have the most potential to identify optimal antibiotic treatment durations. These designs (1) are less vulnerable to allocation concealment bias than the RAR design; (2) are not vulnerable (fixed duration) or are less vulnerable (drop-the-loser) to time-trend bias compared to the RAR or play-the-winner designs; (3) are not associated with the important logistical challenges often accompanying adaptive trials that allow for changes in the allocation ratios (play-the-winner and RAR designs) [32, 43, 44]; and (4) are more likely than the RAR and play-the-winner designs to have sufficient numbers of patients in each arm and/or subgroup at the end of the trial to estimate the complete duration–response curve with sufficient precision, and hence enable evaluation of the potential for important differences in the optimal duration within specific subgroups.

A potential advantage of the drop-the-loser design over the fixed duration design is that the former can drop duration arms that are clearly inferior versus the standard (maximum) duration based on formal statistical analysis. This may ethically be more acceptable by reducing the number of patients allocated to inferior treatment durations.

Although we have only provided theoretical considerations regarding these four designs, we urge the research community to consider developing, testing and applying alternative trial designs that can identify optimal treatment durations, including sample size calculations.

### Extensions

Whilst we have focussed on antibiotic duration, evidence supporting doses of many commonly used antibiotics is similarly scarce, and similar methods could also be used to optimise dose. In practice, particularly in primary care, different durations may well be completely equivalent in terms of acute recovery, yet rare but important complications may vary with different durations. Very large numbers would need to be randomised to estimate ‘duration–response curves’ for these rare outcomes, potentially as co-primary endpoints, or incorporated in a decision analytic framework together with other outcomes [45]. Finally, in the context of changing patterns of resistance or access to care, for example, the optimal duration for any specific indication today may not be optimal tomorrow. A platform duration trial, which allows for the dropping and addition of arms, could be a solution to providing continuously relevant evidence [46], and would also enable different durations of different drugs to be compared.

## Conclusion

There is a clear need for more evidence on optimal antibiotic treatment durations. Multi-arm designs that estimate duration–response curves have a much higher probability of finding the optimal duration for different conditions and patient populations than conventional two-arm RCTs. More research into the properties of alternative RCT designs that can estimate duration–response curves are needed, as well as actual applications of such designs to better identify optimal antibiotic treatment durations. Strengthening the evidence on antibiotic treatment duration is critical in guiding antibiotic stewardship and reducing harm from antibiotic resistance and adverse drug effects.

## Data Availability

Not applicable.

## Abbreviations

DOOR/RADAR: Desirability of Outcome Ranking/Response Adjusted for Duration of Antibiotic Risk
ITT: Intention-to-treat
RAR: Response-adaptive randomisation
RCT: Randomised controlled trial
